# Regulation and Essentiality of the StAR-related Lipid Transfer (START) Domain-containing Phospholipid Transfer Protein PFA0210c in Malaria Parasites*

**DOI:** 10.1074/jbc.M116.740506

**Published:** 2016-10-02

**Authors:** Ross J. Hill, Alessa Ringel, Ellen Knuepfer, Robert W. Moon, Michael J. Blackman, Christiaan van Ooij

**Affiliations:** From the ‡ The Francis Crick Institute, Mill Hill Laboratory, The Ridgeway, Mill Hill, London NW7 1AA and; the Departments of § Infection and Immunity and; ¶ Pathogen Molecular Biology, London School of Hygiene & Tropical Medicine, London WC1E 7HT, United Kingdom

**Keywords:** lipid transport, malaria, parasitology, phospholipid, plasmodium

## Abstract

StAR-related lipid transfer (START) domains are phospholipid- or sterol-binding modules that are present in many proteins. START domain-containing proteins (START proteins) play important functions in eukaryotic cells, including the redistribution of phospholipids to subcellular compartments and delivering sterols to the mitochondrion for steroid synthesis. How the activity of the START domain is regulated remains unknown for most of these proteins. The *Plasmodium falciparum* START protein PFA0210c (PF3D7_0104200) is a broad-spectrum phospholipid transfer protein that is conserved in all sequenced *Plasmodium* species and is most closely related to the mammalian START proteins STARD2 and STARD7. PFA0210c is unusual in that it contains a signal sequence and a PEXEL export motif that together mediate transfer of the protein from the parasite to the host erythrocyte. The protein also contains a C-terminal extension, which is very uncommon among mammalian START proteins. Whereas the biochemical properties of PFA0210c have been characterized, the function of the protein remains unknown. Here, we provide evidence that the unusual C-terminal extension negatively regulates phospholipid transfer activity. Furthermore, we use the genetically tractable *Plasmodium knowlesi* model and recently developed genetic technology in *P. falciparum* to show that the protein is essential for growth of the parasite during the clinically relevant asexual blood stage life cycle. Finally, we show that the regulation of phospholipid transfer by PFA0210c is required *in vivo*, and we identify a potential second regulatory domain. These findings provide insight into a novel mechanism of regulation of phospholipid transfer *in vivo* and may have important implications for the interaction of the malaria parasite with its host cell.

## Introduction

Phospholipid transfer proteins play important roles in the trafficking of phospholipids within eukaryotic cells (1). One subset of phospholipid transfer proteins is represented by a group of proteins containing a StAR-related (START)^4^ lipid-transfer domain, which mediates the binding to lipids or sterols and can promote their transfer between membranes. Although sequence similarity between different START domains can be very low, all are characterized by a specific fold consisting of four α-helices and a nine-stranded twisted antiparallel β-sheet that together form a cavity in which a hydrophobic phospholipid or sterol is held (2, 3). The human genome encodes 15 different START domain-containing proteins (START proteins) that can be categorized into five different groups based on the bound ligand specificity and the presence of additional functional domains (Table 1) (4–7). The roles of the different START proteins are in most cases not well understood, although mutations in genes encoding START proteins have been linked with various diseases (5, 8). It is clear from genetic experiments in mice that at least two of the murine START proteins, STARD11 and STARD12, are essential (9, 10). The best characterized human START proteins are STARD2, STARD7, and STARD10, which all transfer phosphatidylcholine (and additionally phosphatidylethanolamine in the case of STARD10) (11–13). Although their exact functions are unclear, the proteins appear to have a role in the transfer of phospholipids from the endoplasmic reticulum to mitochondria and possibly the plasma membrane (14, 15). What is also not understood is whether and how the transfer of phospholipids by these proteins is regulated. Some of the START proteins consist of little more than the START domain itself, whereas others contain additional domains, such as thioesterase or Rho-GAP domains (Table 1). Some START proteins, including STARD2 and STARD12, have been shown to interact with other proteins (16, 17), which may provide a mechanism of regulating phospholipid transfer activity, or conversely, to allow the START protein to regulate the interacting protein. In the case of STARD10, phosphorylation of a residue in a C-terminal extension has been shown to regulate transfer activity (18).

Most members of the genus *Plasmodium*, which are the obligate intracellular parasites that cause malaria, encode four START proteins (19). These include a putative orthologue of STARD2 (phosphatidylcholine transfer protein; PF3D7_1351000) and an uncharacterized protein (PF3D7_0911100) with a putative cyclin-dependent serine/threonine kinase domain that also contains a START domain at its C terminus. The third START protein (PF3D7_1463500) displays similarity to StarD3 (MLN64), a cholesterol transfer protein (20). Interestingly, in rodent malaria parasites this protein forms part of the large Fam A family, whereas in non-rodent malaria parasites, only one family member is present (21–23). The best characterized START protein in *Plasmodium* spp. is the exported broad specificity phospholipid transfer protein PFA0210c (PF3D7_0104200). PSI-BLAST analysis shows that PFA0210c is most closely related to STARD7, whereas structure prediction reveals that the highest similarity is to human phosphatidylcholine transfer protein STARD2 (24).

**FIGURE 1.**
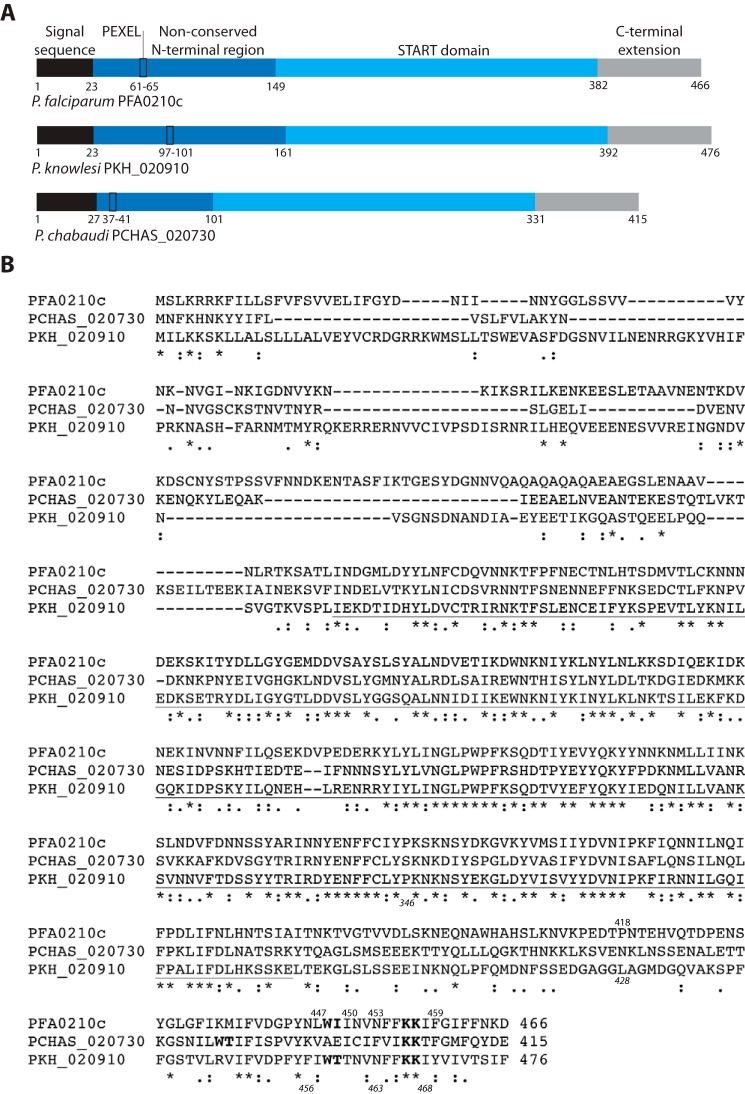
**Overview and alignment of the PFA0210c orthologues from *P. falciparum*, *P. knowlesi* (PKH_020910), and *P. chabaudi* (PCHAS_020730).** *A,* outline of the domains of PFA0210c and its orthologues, indicating the signal sequence (*black*), the motif that mediates export to the erythrocyte (PEXEL; *black rectangle*), the non-conserved N-terminal region (*dark blue*), the START domain (*light blue*), and the C-terminal extension (*gray*). *Numbers* above the outline indicate the position of the amino acid residues at the start and end of the domains. The domain structure among the PFA0210c orthologues is the same, although the length of the non-conserved N-terminal domain, and therefore the length of the entire protein, varies. *B,* alignment of PFA0210c with its orthologues of *P. knowlesi* (PKH_020910) and *P. chabaudi* (PCHAS_020730). The START domain is *underlined*. Conserved residues in the C terminus that were targeted in the mutagenesis studies are shown in *boldface type*. The *numbers* at the end of the sequence indicate the position of the residue at the extreme C terminus; the *numbers* above the sequence and the *italicized numbers* below the sequence indicate the position of the last residue of the truncations of the *P. falciparum* and *P. knowlesi* protein, respectively, used in this study. Note that the variation in sequence length is determined by variation in the N-terminal portion of the proteins; the length of the sequence from the start of the START domain to the C terminus varies by only two residues between these proteins. Sequences were obtained from PlasmoDB (40) and aligned using Clustal Omega (45).

Phospholipid transfer proteins in *Plasmodium* are of particular interest as one of the most striking changes induced by the parasite in the host erythrocyte, the site of replication of the parasite during the clinical stage of the disease, is the formation of a large exomembrane system (25). This consists of several different, most likely unconnected, membranous compartments that have various functions within the cell. One of these membranous compartments is the parasitophorous vacuole membrane that surrounds the parasite during the entire intraerythrocytic life cycle and that separates the parasite from the erythrocyte cytosol. Another group of membranous compartments is the Maurer's clefts, small vesicles that are important for the transfer of parasite proteins to the surface of the infected cell (26, 27). As mature erythrocytes are devoid of internal membranes and lack the capacity to produce membranes (28), these newly formed membranes, which are all outside of and unconnected to the parasite, must be produced by the parasite. How the parasite transfers phospholipids across the aqueous environment of the parasitophorous vacuole lumen to the parasitophorous membrane and the Maurer's clefts is unknown. It has been suggested that PFA0210c may play a part in this process (24). In support of this, a unique feature of PFA0210c among the *Plasmodium falciparum* START proteins is the presence of a signal sequence, which mediates the secretion of the protein from the parasite into the parasitophorous vacuole, as well as a PEXEL export signal, which directs export beyond the parasitophorous vacuole membrane into the erythrocyte cytosol (Fig. 1*A*). Previous studies have indicated that PFA0210c can be exported from the parasite to the erythrocyte cytosol (29), although at least a fraction remains in the parasitophorous vacuole (24). The presence of the protein at these locations indicates that it may function to transfer phospholipids between the parasite and parts of the exomembrane system.

Here, we show that PFA0210c and its *Plasmodium* orthologues contain an unusual C-terminal extension that regulates the phospholipid transfer activity of the protein. Furthermore, we provide evidence that the *Plasmodium knowlesi* orthologue of PFA0210c, PKH_020910, is essential, and we apply new genetic techniques to show that *P. falciparum* parasites lacking PFA0210c do not develop or proliferate within the infected erythrocyte. Additionally, we show that the regulation of phospholipid transfer through the C-terminal extension is required for parasite growth. Together, these experiments reveal a new mechanism of regulating phospholipid transfer and show that phospholipid transfer is an essential and regulated process in *Plasmodium* parasites.

## Results

### 

#### 

##### PFA0210c and Its Orthologue in P. knowlesi Are Essential

**FIGURE 2.**
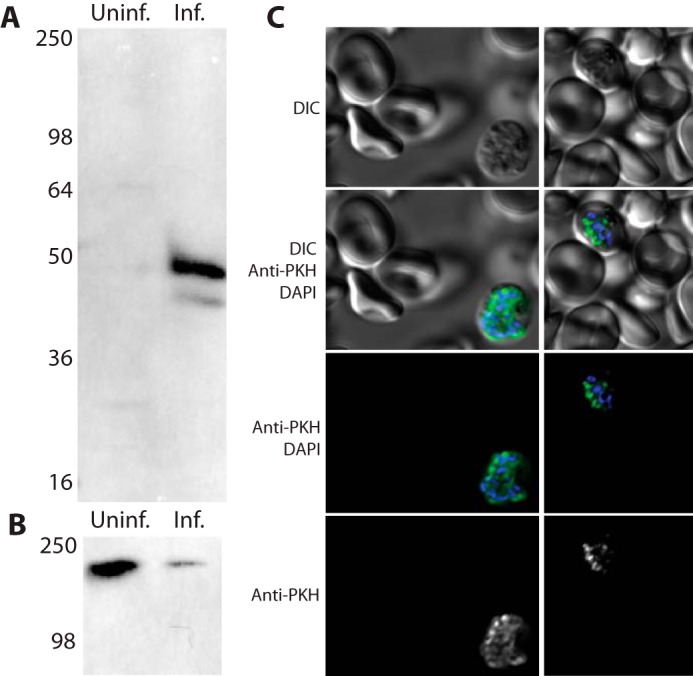
**PKH_020910 is produced during the asexual intraerythrocytic life cycle.** *A,* immunoblot using anti-PKH_020910 antiserum probing extracts of either uninfected erythrocytes (*Uninf.*) or erythrocytes infected with *P. knowlesi* (*Inf.*). *B,* immunoblot using anti-spectrin antibodies of the same extracts as in *A*. The more intense band in the uninfected lane indicates that more cell equivalents were loaded in the lane containing the extract of uninfected erythrocytes. *C,* detection of PKH_020910 in infected erythrocytes by indirect immunofluorescence microscopy. Erythrocytes infected with late-stage *P. knowlesi* were stained with anti-PKH_020910 antiserum and DAPI to visualize the parasite nuclei. Staining is clearly visible within the parasites but is absent in uninfected erythrocytes. *DIC,* differential interference contrast.

PFA0210c was initially identified as a conserved exported protein (29). We subsequently showed that PFA0210c as well as its orthologues in the simian and human malaria pathogen *P. knowlesi* (PKH_020910 or PKNH_0209300) and the rodent parasite *Plasmodium chabaudi* (PCHAS_020730 or PCHAS_0207300) are phospholipid transfer proteins that can transfer a broad range of phospholipids *in vitro* (24). All three proteins contain a signal sequence and a predicted PEXEL motif that directs export from the parasite into the host erythrocyte (Fig. 1*A*) (29–31). The proteins are characterized by a poorly conserved N-terminal region, a START domain that is required for the phospholipid transfer activity (24), and a C-terminal extension of ∼84 amino acid residues (Fig. 1*B*). To gain further insight into the function of this protein, we used the recently developed *P. knowlesi* A.1-H.1 strain that has been adapted to *in vitro* growth in human erythrocytes (32) to attempt to generate a mutant that lacks the gene encoding PKH_020910. The *P. knowlesi* model is highly genetically tractable, allowing for rapid gene modification by homologous recombination. We first confirmed that PKH_020910 is expressed in *in vitro* culture by immunoblotting. Full-length PKH_020910 has a predicted molecular mass of 55.4 kDa, which is reduced to 52.7 kDa after removal of the signal sequence and further reduced to 43.7 kDa after cleavage of the PEXEL domain. Using an affinity-purified polyclonal antibody, a protein of approximately the expected molecular mass was detectable in extracts of infected erythrocytes (Fig. 2*A*) but not in that of uninfected erythrocytes, despite the presence of a higher amount of protein in the lane with uninfected erythrocyte extract (Fig. 2*B*). Immunofluorescence staining of erythrocytes infected with late stage parasites (schizonts) revealed that the protein is likely expressed in a subset of apical organelles, as indicated by the localized punctate pattern of staining (Fig. 2*C*). The identity of this organelle could not be ascertained because of the lack of organelle markers for *P. knowlesi*, and no signal was obtained by immunoelectron microscopy using the available anti-PKH_020910 antibodies. Nonetheless, these experiments confirmed that PKH_020910 is expressed in blood stages of the *P. knowlesi* life cycle.

**FIGURE 3.**
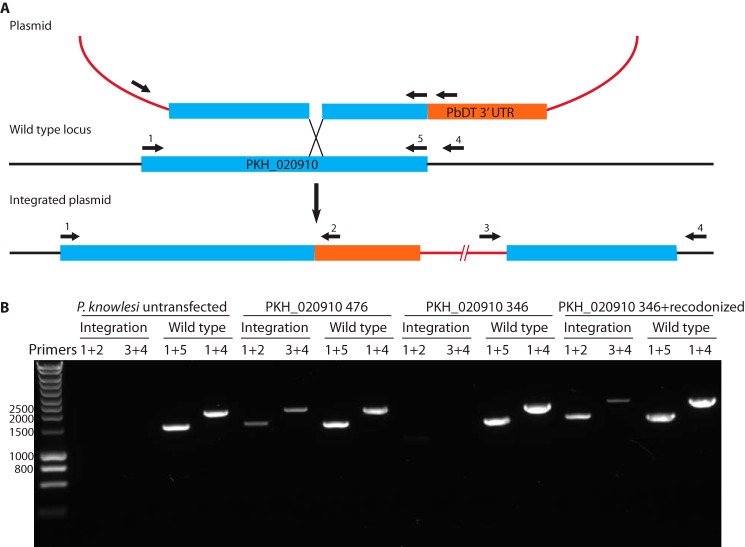
**PKH_020910 is essential in *P. knowlesi*.** *A,* outline of the transfection strategy and location of the primers used for the verification of integration of the plasmids. The plasmid containing the fragment of PKH_020910 was linearized with BsaBI within the PKH_020910 coding sequence to promote recombination and prevent plasmid propagation. Note that the 5′ region of PKH_020910 that encodes the signal sequence was omitted from the integration region. The duplicated copy of *PKH_020910* produced following integration therefore lacks a start codon and cannot give rise to an exported protein. The plasmid backbone is shown in *purple. B,* verification of integration of the *PKH_020910* targeting plasmids using the primers shown in *A*. Integration-specific products were detected only with DNA from parasites transfected with targeting vectors that would reconstitute the entire gene (*PKH_020910 476* and *PKH_020910 428*+*recodonized*) and not with the targeting vector designed to truncate the gene to produce a non-functional protein or with DNA from untransfected parasites. The *number* after the gene name indicates the last codon of *PKH_020910* in the targeting region. Expected sizes of the PCR products are as follows: *1*+*2,*1469 bp; *3*+*4*, 1919 bp; *1*+*5*, 1453 bp; *1*+*4*, 1892 bp. Sizes of relevant standards are indicated on the *left-hand side*. All transfections were repeated a minimum of three times, each time yielding the same result.

To determine whether PKH_020910 is required for parasite growth, we attempted to disrupt the *PKH_020910* gene through single crossover homologous recombination (Fig. 3*A*). Parasites were transfected with linearized plasmids that were designed to integrate into the genome and either reconstitute the entire gene or truncate the coding sequence to remove 46 residues of the START domain. A similar truncation of PFA0210c ablates its capacity to transfer phospholipids in an *in vitro* assay (24). After selection of the transfected parasites with pyrimethamine, drug-resistant parasites were recovered in all cases within 10–14 days. Diagnostic polymerase chain reaction (PCR) analysis of genomic DNA (Fig. 3*A*) indicated that integration of the plasmid could only be detected in the case of the plasmids designed to reconstitute the entire open reading frame (*PKH_020910 476* in Fig. 3*B*); no integration was detected in the plasmid designed to truncate the gene (*PKH_020910 346* in Fig. 3*B*). To rule out the unlikely scenario that the different results reflected the different lengths of the targeting region within the plasmids (972 bp *versus* 1362 bp), we engineered a plasmid that contained the same 972-bp homology region as the deletion plasmid but also contained the remainder of the entire gene in a re-codonized form. Integration of this plasmid was thus mediated by the same targeting sequence as the truncation plasmid but would lead to reconstitution of the full-length gene. This plasmid readily integrated into the chromosome (*PKH_020910 346*+*recodonized* in Fig. 3*B*), indicating that the inability of the truncation to integrate was not a consequence of the insufficient targeting sequence but rather reflected a requirement for PKH_020910 for parasite viability.

**FIGURE 4.**
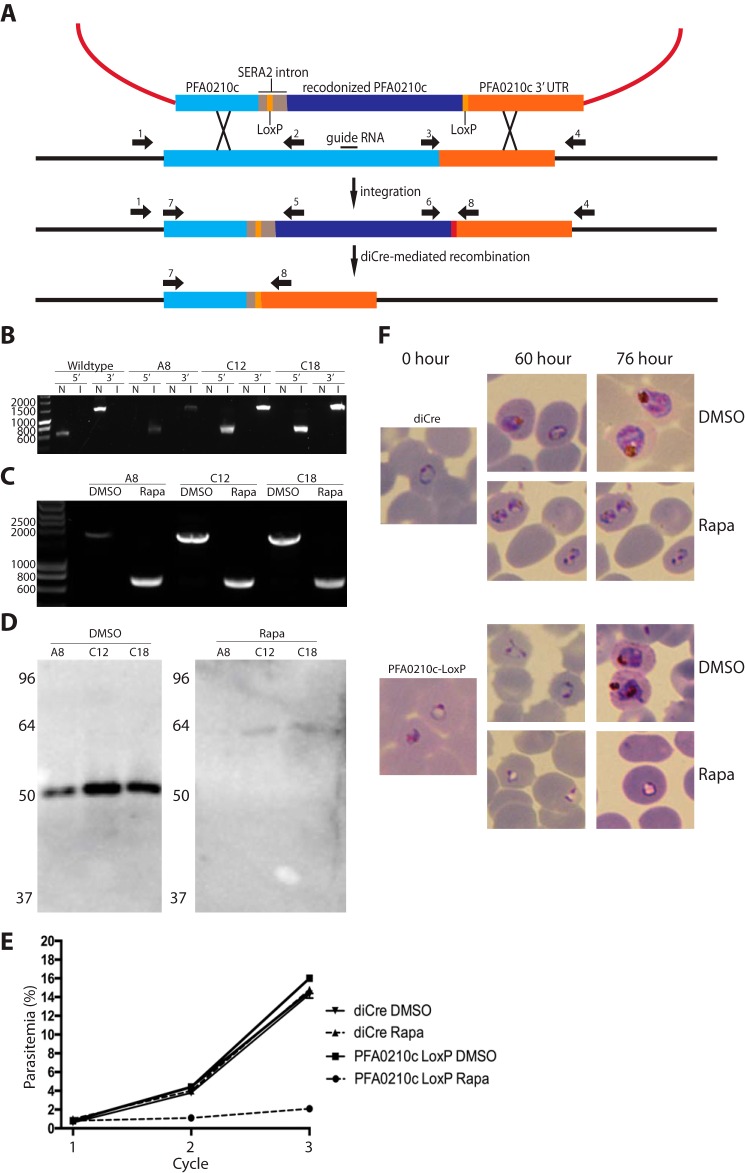
**Inducible removal of PFA0210c in *P. falciparum* leads to rapid death of the parasite.** *A,* integration strategy for the replacement of the native PFA0210c locus with a version of the gene containing two *loxP* sites. Three separate guide RNAs were used to facilitate integration of into the native gene. Induction of Cre recombinase activity by the addition of rapamycin induces the excision of the sequence between the two *loxP* sites. *B,* diagnostic PCR of wild type *P. falciparum* and PFA0210c-LoxP clones A8, C12, and C18 using primers specific for either the native sequence or the integrated re-codonized sequence. Primers used are CVO150 (*1*) and CVO083 (*2*) (*5′ N*), CVO150 and CVO162 (*5*) (*5′ I*), CVO071 (*3*) and CVO183 (*4*) (*3′ N*), and CVO321 (*6*) and CVO183 (*3′ I*). *N* indicates primer pair specific for native locus, and *I* indicates primer pair specific for the integrated PFA0210-LoxP. *C,* verification of excision after addition of rapamycin in clones A8, C12, and C18. Parasites were treated with 10 nm rapamycin or an equivalent volume of DMSO for 1 h. Forty hours later genomic DNA was extracted and used as template for PCR with primers 7 (CVO001) and 8 (CVO097). Successful excision decreases the size of the expected band from 1536 to 561 bp. *D,* immunoblot using anti-PFA0210c antibodies shows that rapamycin (*Rapa*)-treated parasites lack PFA0210c. Parasites were treated early in the ring stage, and proteins were extracted ∼40 h later. *E,* growth rate of the parasites lacking PFA0210c is severely decreased. Parasites containing *PFA0210c-LoxP* or the DiCre-expressing parent clone were synchronized in the early ring stage and diluted to a parasitemia of ∼0.6%. The parasites were then treated with 10 nm rapamycin or the equivalent volume of DMSO for 1 h. Parasitemia was measured immediately before treatment (cycle 1), 76 h after diluting (cycle 2), and 96 h after diluting. Parasite cultures were set up in triplicate. *Error bars* indicate standard deviation. *F,* parasites lacking PFA0210c form aberrant ring-stage parasites and do not develop past the ring stage. Parasites used in *E* were used to make Giemsa-stained smears at the times indicated. Parasites from the parent clone appear normal, as do the DMSO-treated PFA0210c-LoxP parasites. PFA0210c-LoxP parasites treated with rapamycin form small rings with vacuolated centers. At the 76-h time point, these parasites have not developed further, in contrast to the parent strain or the DMSO-treated PFA0210c-LoxP strain.

To avoid caveats associated with negative data, we wanted to confirm further that this protein is required for parasite survival. We exploited a combination of recently developed Cas9 technology with conditional gene excision (33–36) to obtain an inducible disruption of the gene in order to examine the essentiality of PFA0210c in *P. falciparum*. For this, we replaced the native PFA0210c open reading frame with a form interrupted by a *loxP*-containing intron (37). This was followed by 855 bp of a re-codonized *PFA0210c*-coding sequence plus a second *loxP* site immediately following the stop codon (Fig. 4*A*). This gene modification was performed in the 1G5DC *P. falciparum* clone (38), which expresses the rapamycin-inducible Cre recombinase (DiCre) (38, 39). Several transgenic parasite clones were generated expressing the *loxP*-containing *PFA0210c* gene (called PFA0210c-LoxP; Fig. 4*B*). Treatment of these clones with rapamycin resulted in the expected excision of the segment of the *PFA0210c* gene that is flanked by the *loxP* sites (Fig. 4*C*). In confirmation of this, no PFA0210c protein could be detected in schizonts of the rapamycin-treated parasites (Fig. 4*D*). To assess the effects of gene disruption on parasite viability, growth assays were performed, comparing rapamycin-treated PFA0210c-LoxP parasites with control mock-treated counterparts. This revealed a severe growth defect in the parasites lacking PFA0210c (Fig. 4*E*), whereas treatment of 1G5 parasites with rapamycin did not affect their growth rate. Giemsa staining of the parasites revealed that loss of PFA0210c resulted in the formation of dysmorphic ring-stage parasites in the growth cycle following that in which the parasites were treated with rapamycin (the 60-h time point in Fig. 4*F*). These parasites have a translucent, nearly white center and do not develop past the ring stage, whereas DMSO-treated parasites and 1G5 parasites do develop into trophozoites (see the 76-h time point in Fig. 4*F*). These results demonstrate that PKH_020910 and PFA0210c are essential proteins in the asexual blood stage of the *Plasmodium* life cycle.

##### PFA0210c and Its Orthologues Contain an Extended C Terminus

The 84-residue C-terminal extension of PFA0210c is highly unusual among START proteins. Although the 15 human START proteins range in size from 205 residues (STARD5) to 4705 residues (STARD9), the START domain is almost exclusively located at or very near the extreme C terminus of the protein (Table 1); the longest C-terminal extension found in a human START protein, STARD10, is 50 residues. All orthologues of PFA0210c possess a C-terminal extension ranging from 81 residues in the *Plasmodium yoelii* orthologue (PY17X_0210300) to 92 residues in the *Plasmodium berghei* orthologue (PBANKA_0208900), with most (including those of PFA0210c, PKH_020910, and PCHAS_020730) comprising 84 residues (Table 1)). Sequence conservation in the C-terminal extension is overall much lower than within the START domain (Fig. 1*B*), However, we noticed the presence of a number of highly conserved residues near the extreme C terminus, suggesting a conserved function (Fig. 1*B*).

**TABLE 1:** **Location of START domains in human START-domain-containing proteins and PFA0210c orthologues** Information was obtained from the NCBI protein resource. Proteins are grouped as described (5, 6).

Family	Name	Length (amino acids)	START domain (amino acids)	Extension
**Human**				
Cholesterol/sterol carrier	StarD1 (StAR)	285	67–280	5
	StarD3*^a^*	445	233–441	4
	StarD4*^b^*	205	5–205	0
	StarD5	213	6–211	2
	StarD6	220	1–204	16
Sphingolipid/glycerolipid carriers	StarD2 (PCTP)	214	4–210	4
	StarD7*^c^*	370	117–325	45
	StarD7*^d^*	269	16–224	45
	StarD10	291	19–241	50
	StarD11 (CERT)	598	364–598	0
Rho-GAP START domains	StarD12 (DLC-1)*^e^*	1528	1315–1518	10
	StarD13 (DLC-2)*^f^*	1124	911–1115	9
	StarD8*^g^*	1107	894–1098	9
Thioesterase START domains	StarD14 (BFIT)	607	344–583	24
	StarD15 (CACH)	555	311–546	9
Other	StarD9*^h^*	4705	4496–4705	0

***Plasmodium* spp**.				
*P. falciparum*	PFA0210c	466	149–382	84
*P. chabaudi*	PCHAS_020730	415	101–331	84
*P. knowlesi*	PKH_020910	476	161–392	84
*P. berghei*	PBANKA_0208900	441	118–349	92
*P. yoelii*	PY17X_0210300	431	119–350	81
*P. vivax*	PVX_081550	495	179–410	85

*^a^* This is isoform 1.

*^b^* This is isoform a.

*^c^* This is isoform CRA_a.

*^d^* This is mitochondrial isoform X1.

*^e^* This is isoform 1.

*^f^* This is isoform X2.

*^g^* This is isoform X2.

*^h^* This is isoform X1

##### C Terminus of PFA0210c Negatively Regulates Phospholipid Transfer

**FIGURE 5.**
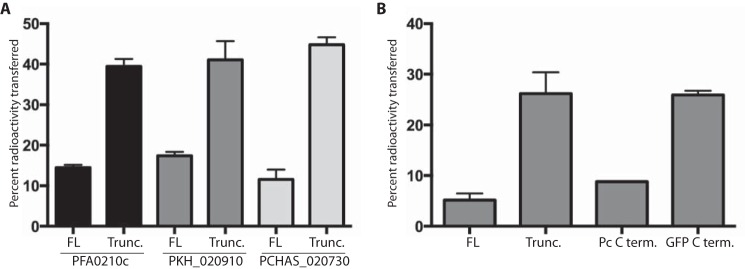
**C-terminal extension of PFA0210c and its orthologues regulates phospholipid transfer.** *A,* phospholipid transfer activity of full-length (*FL*) and truncated (*Trunc.*) PFA0210c, PKH_020910, and PCHAS_020730. Phospholipid transfer was measured using an activity assay that measures the transfer of radiolabeled phosphatidylcholine from a small population of donor vesicles to a larger population of acceptor vesicles. *B,* phospholipid transfer activity of full-length PFA0210c (*FL*), truncated PFA0210c (*Trunc.*), and PFA0210c where the last 48 amino acid residues have been replaced with the corresponding 48 residues from PCHAS_020730 (*Pc C term.*) or GFP (*GFP-C term.*). All assays were performed in triplicate. *Error bars* indicate standard deviation.

To determine whether the C-terminal extension of PFA0210c has a role in the regulation of its phospholipid transfer activity, we produced recombinant forms of PFA0210c, PKH_020910, and PCHAS_020730 that contained the entire START domain but were truncated to various extents within the C-terminal extension. The proteins were then compared in an *in vitro* phospholipid transfer assay. All the truncated proteins showed higher phospholipid transfer activity than the full-length protein, indicating that the C-terminal extension affects the activity (Fig. 5*A*).

To investigate whether the observed regulation through the C terminus was mediated simply through steric hindrance by a large protein domain or whether it required the presence of a conserved sequence-specific element in the C terminus, we replaced the C-terminal 48 residues of PFA0210c (the residues absent from the truncated version of the protein used in Fig. 4*A*) with either the corresponding sequence of the *P. chabaudi* orthologue or the C-terminal 48 residues of green fluorescent protein (GFP). The chimeric protein containing C-terminal residues of the *P. chabaudi* orthologue displayed levels of phospholipid transfer activity similar to that of full-length wild type PFA0210c, whereas the form containing the GFP sequence showed increased activity, similar to that of the truncated PFA0210c (Fig. 5*B*). These results suggest that regulation of phospholipid transfer activity involves conserved sequence elements in the C-terminal extension. We conclude from these experiments that the C-terminal extension of PFA0210c and its orthologues contains one or more conserved sequence-specific elements that regulate the phospholipid transfer activity of the proteins.

##### Regulation through the C Terminus Is Confined to a Short Conserved Region

**FIGURE 6.**
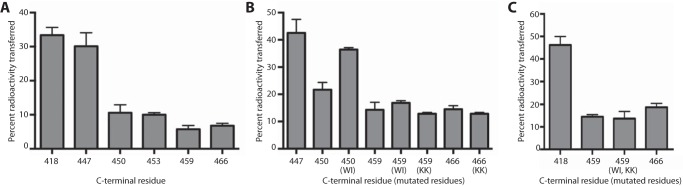
**Small conserved region in the C terminus regulates phospholipid transfer.** *A,* phospholipid transfer activity of truncated forms of PFA0210c *in vitro. Numbers* indicate the C-terminal residue in the truncations. *B,* phospholipid transfer activity of point mutations in truncated proteins and full-length mutations. Indicated are the C terminus and in *parentheses* the point mutations (see Fig. 1*B* for location of mutated residues). *C,* phospholipid transfer activity of a double mutant and control proteins. All assays were performed in triplicate. *Error bars* indicate standard deviation.

We next wanted to define in more detail the region of the C terminus that mediates the regulation of phospholipid transfer. As the sequence conservation is primarily restricted to the C-terminal 27 residues of the protein (Fig. 1*B*), we focused on this region. To identify the residues involved in the regulation of phospholipid transfer activity, we produced serially truncated versions of recombinant PFA0210c that sequentially removed three to six residues (see Fig. 1*B* for the positions where the proteins were truncated) and evaluated the effects on *in vitro* phospholipid transfer activity. This revealed that removal of the last 19 residues (C-terminal residue 447), which includes the highly conserved residues from position 448 through 466, produced fully active protein (Fig. 6*A*). Truncated proteins that lacked between 16 and 13 residues (C-terminal residues 450 and 453, respectively) displayed low activity that was nonetheless higher than that of the full-length protein. A truncation of only seven residues (C-terminal residue 459) had no effect on activity relative to the full-length protein (C-terminal residue 466).

These results show that the phospholipid transfer activity of PFA0210c is regulated through a short patch of residues near the extreme C terminus, extending from Trp-448 to Lys-457. These residues are conserved in *P. falciparum* and *P. knowlesi* but not in the rodent malaria species (Fig. 1*B*). However, the Ile-448 and Trp-449 as well as the Lys residue nine residues downstream that are all part of the conserved region in PFA0210c are also present in the rodent malaria parasite species, albeit shifted by 12 residues compared with the non-rodent malaria parasites. This suggests that the positioning of the regulatory region is shifted slightly in the rodent malaria parasites.

To understand the roles of the conserved residues in more detail, we separately substituted the conserved Trp-448 and Ile-449 residues as well as Lys-456 and Lys-457 in PFA0210c (see *boldface* residues in Fig. 1*B*) into full-length and truncated recombinant proteins. Phospholipid transfer analysis of these mutants revealed that substitution of Trp-448 and Ile-449 in the protein truncated at residue 450 resulted in gain-of-transfer activity, producing activity similar to that of the protein truncated at residue 447. However, the same mutation in the protein that contains an additional nine residues (C-terminal residue 459) did not result in an increase in activity. Mutation of Lys-456 and Lys-457 did not affect phospholipid transfer activity of either full-length or truncated proteins.

To test whether the two Lys residues by themselves provide the additional regulation in proteins lacking the Trp-448 and Ile-449 residues, we produced a mutant protein that contained both sets of mutations (this mutant also lacks the last seven residues, as full-length protein with the Trp-448 and Ile-449 mutation could not be obtained in a non-aggregated form). However, this double mutant protein did not display enhanced activity above that of the wild type protein.

Together, these results show that the phospholipid transfer activity of PFA0210c is regulated by specific residues in the extreme C-terminal 18-residue segment of the protein. The Trp-448 and Ile-449 residues play a pivotal role in this regulation, but additional residues are also involved.

##### Regulation of Phospholipid Transfer Activity Is Required in Vivo

**FIGURE 7.**
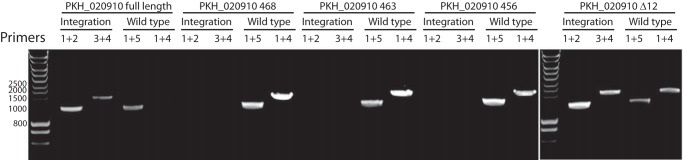
**Regulation of phospholipid transfer is required *in vivo*.** Diagnostic PCR analysis of attempted integration of truncation plasmids designed to remove 8 codons (PKH_020910 468), 13 codons (PKH_020910 463), or 20 codons (PKH_020910 456) from the end of the gene encoding PKH_020910 or to remove internal codons 433–444 (PKH_020910 Δ12). Integration-specific PCR products were detected only in parasites that reconstituted the entire gene (PKH_020910 full length) and PKH_020910 Δ12. The PCR strategy and the primers used are described in Fig. 3*A*. All transfections were repeated a minimum of three times, each time yielding the same result.

Having shown that the phospholipid transfer protein is required for growth of the parasite, we next wanted to determine whether the regulation of phospholipid transfer revealed through the *in vitro* experiments is functionally important *in vivo*. For this, we used the same integration strategy as described in Fig. 3*A* to attempt to introduce serial truncations of *PKH_020910* in the *P. knowlesi* genome. Integration of the various targeting plasmids used was designed to produce truncated genes encoding proteins lacking the C-terminal eight residues (retaining all the residues required for the regulation of the phospholipid transfer activity *in vitro*), 13 residues (thereby removing some but not all of the regulatory residues), or 20 residues (thereby removing all of the regulatory residues). In addition, we used a plasmid that upon integration would produce a gene that encodes a protein lacking internal residues 433–444 of the C-terminal extension (Δ12), thus shortening the C-terminal extension but retaining the regulatory residues at the extreme C terminus. Parasites were recovered after transfection with each plasmid, and integration was determined using the same strategy as described in Fig. 1. This revealed that the positive control plasmid designed to reconstitute the entire gene readily integrated (Fig. 7). However, integration of none of the plasmids designed to give rise to a truncated protein was detected. These results indicate that regulation of phospholipid transfer by the protein is required for parasite growth.

Surprisingly, the plasmid designed to give rise to a protein lacking 12 residues of the C-terminal extension but containing the conserved region at the end of the C terminus readily integrated. It thus appears that the residues at the extreme C terminus of PKH_020910 are required for regulation but that their distance from the end of the START domain may vary.

## Discussion

In this work, we have shown that the START protein PFA0210c and its orthologues are required for the growth of the malaria parasite in its clinically relevant blood stages. Replacement of the native gene with a version that encodes a protein that cannot transfer phospholipids was not successful in *P. knowlesi*, whereas control plasmids readily integrated, strongly suggesting that parasites lacking a functional gene are not viable. Furthermore, conditional disruption of the PFA0210c gene in *P. falciparum* blocked the development of the parasites in the subsequent round of replication. Further support for the essential nature of the protein is that genetic modification of the orthologous gene in *P. berghei* was unsuccessful, as listed on PlasmoDB (www.plasmodb.org (40)).

Additional *in vitro* analysis showed that PFA0210c is regulated through a unique C-terminal extension. The residues responsible for the regulation lie close to the extreme C terminus and are conserved among the *Plasmodium* orthologues. This conservation of sequence is supported by the demonstration that the corresponding *P. chabaudi* sequence could restore regulation of the *P. falciparum* orthologue PFA0210c *in vitro*, whereas replacement of the C terminus with the equivalent sequence from GFP did not affect the regulation. In further experiments, the presence of the C-terminal 48 residues of PFA0210c in the form of a separate peptide or a glutathione *S*-transferase (GST) fusion protein did not affect phospholipid transfer levels of truncated PFA0210c, indicating that the regulatory function of the C-terminal sequence cannot be mediated in *trans*, although we cannot rule out that the GST fusion and the peptide were not in the correct conformation to mediate their effect. Importantly, this regulation is likely to be essential for parasite viability, as we were unable to obtain *P. knowlesi* parasites expressing a truncated version of the protein. The precise location of the regulatory region relative to the end of the START domain appears to be somewhat flexible, as replacing the native gene with a version that encodes a protein in which the extreme C terminus is shifted 12 residues closer to the START domain appeared to be tolerated *in vivo*. Consistent with this, in the rodent malaria parasite orthologues, residues that are highly conserved in the non-rodent malaria parasites, are shifted by 12 residues. Interestingly, we could not obtain viable parasites that expressed a version of the protein that lacked the C-terminal eight amino acids but retained the residues that are important for regulating phospholipid transfer *in vitro*. Hence, *in vivo* the regulation of phospholipid transfer by PFA0210c and its orthologues may be more complex. We hypothesize that there are two regulatory regions as follows: the first region bounded by the Trp-448 and Ile-449 and the Lys residues at position 456 and 457 that was identified in the *in vitro* assay as the negative regulatory region (the WI-KK domain); and a second region, closer to the C terminus, that can counteract the negative regulation mediated by the WI-KK domain upon a signal from another source. Hence, removing this second region would make the protein permanently inactive, as the regulation mediated by the WI-KK domain cannot be removed. The protein is essential for the growth of the parasite, and therefore, removing its capacity to activate phospholipid transfer activity would be a lethal event for the parasite. Potentially, a cofactor is required that interacts with the second region to counteract the negative regulation of the WI-KK domain in the transfer of phospholipids. This would allow for the intriguing possibility that this cofactor regulates the directionality of phospholipid transfer; the protein would only be able to obtain and/or release its phospholipid to a membrane in which the cofactor is present. Identification of a binding partner of PFA0210c would shed light on this model.

This report is only the second to provide evidence for regulation of the activity of a mammalian START protein. The only other example is that of STARD10, which similarly contains a C-terminal extension, albeit shorter (50 amino acids compared with 86 amino acids in PFA0210c; Table 1). This C-terminal extension is phosphorylated *in vivo*, whereas phosphorylation *in vitro* with casein kinase II decreased its phospholipid transfer activity (18). Interestingly, an eight-amino acid truncation of STARD10, removing the phosphorylation site, increased its activity beyond that of the unphosphorylated protein. It was initially speculated that phosphorylation of the protein may decrease the binding of STARD10 to membranes, but in light of the results presented here, it may also be possible that phosphorylation of the protein induces a conformational shift that reduces activity. This may point to a broader regulation of START proteins that contain a C-terminal extension. In conjunction with the previous report (18), this study begins to elucidate a mechanism by which phospholipid transfer can be regulated *in vivo*, a mechanism potentially shared from Apicomplexa to humans.

## Experimental Procedures

### 

#### 

##### Parasite Culture and Transfection

The human-adapted *P. knowlesi* clone A.1-H.1 was cultured as described (32). Briefly, parasites were maintained in human erythrocytes (2% hematocrit) in RPMI 1640 medium supplemented with 2.3 g/liter sodium bicarbonate, 4 g/liter dextrose, 5.957 g/liter HEPES, 0.05 g/liter hypoxanthine, 5 g/liter Albumax II, 0.025 g/liter gentamycin sulfate, 0.292 g/liter l-glutamine, and 10% (v/v) horse serum.

For transfections of *P. knowlesi*, parasites were transfected using the Amaxa 4D electroporator (Lonza), as described previously (32, 41). Late stage parasites were harvested by flotation on a 55% Nycodenz (Axis-Shield) stock solution (consisting of 27.6% (w/v) Nycodenz powder in RPMI 1640 medium). Purified parasites were maintained in RPMI 1640 medium until a majority had reached the eight-nuclei stage. The parasites were then centrifuged briefly, and the supernatant was removed. The parasites were suspended in 100 μl of nucleofection solution (P3 Primary cell 4D Nucleofector X Kit L (Lonza)), and 10 μl of TE containing ∼50 μg of plasmid that had been linearized with BsaBI was added, and the parasites were subsequently electroporated. Parasites were transferred to a flask containing 300 μl of blood and 1.7 ml of RPMI 1640 medium and maintained, with shaking, for 30 min at 37 °C to allow for efficient invasion. Eight ml of medium was added, and the parasites were further maintained at 37 °C. Selection for transfected parasites with 0.1 μm pyrimethamine was initiated ∼20 h after transfection. Drug-resistant parasites were usually detected 10–14 days later.

Integration of plasmids in *P. knowlesi* was determined by isolating genomic DNA from the parasites using the Qiagen Blood and Tissue kit and using the extracted DNA as template for PCR using primer pairs specific for the integrated plasmid (CVO093 and CVO079, M13 reverse, and CVO104), the wild type locus (CVO093 and CVO104), and circularized plasmid (M13 reverse and CVO079) as described under “Results.” All primer sequences are listed in Table 2. The 1G5DC clone and the PFA0210c-LoxP strain of *P. falciparum* 3D7 were maintained as *P. knowlesi*, without the addition of human serum.

**TABLE 2:** **Primers used in this study** Restriction sites are shown in lowercase.

CVO010	GGACctcgagTCAGAAGATGCTGGTAACGATAACGTAAATTTTTTTG
CVO014	GGACgaattcGTCAGAGAAATAAACGGAAATGATG
CVO015	GGACctcgagTCAGTGATGGTGATGGTGATGGAAGATGCTGGTAACGATAACGTAAATTTTTTTG
CVO021	GGACctcgagTCAGTGATGGTGATGGTGATGTGGAGTATCTTCTGGCTTAACGTTTTTTAAACTATGTG
CVO022	GGACgaattcAAAAGTGCAACTTTAATAAATGATGGTATGTTAGAT
CVO054	GGACctcgagTCAGTGATGGTGATGGTGATGATAAGAATTTTCTGGATCCGTTTGTAC
CVO057	GGACctcgagTCAGTGATGGTGATGGTGATGAAATATTTTTTTAAAAAAGTTAACGTTGATGATCC
CVO059	GGACtctagaAAGGAAAACCAAAAATATTTAGAACAAGCTAAG
CVO060	GGACctcgagTCAGTGATGGTGATGGTGATGTTCATCATATTGGAACATACCAAATG
CVO061	GGACctcgagTCAGTGATGGTGATGGTGATGATTTTCGACTGATTTAAGTTTTTTATTATGGG
CVO064	GGACctcgagTTAGTGATGGTGATGGTGATGTCCCGCCAATCCACCAGC
CVO065	CTAAAGCGTTTTCTGAACTATTTAATTTTGGAGTATCTTCTGGCTTAACGTTTTTTAAACTATGTG
CVO066	CACATAGTTTAAAAAACGTTAAGCCAGAAGATACTCCAAAATTAAATAGTTCAGAAAACGCTTTAG
CVO067	CTTAAATAATGATTATCTGGTAATAATACAGGTCCTGGAGTATCTTCTGGCTTAACGTTTTTTAAACTATGTG
CVO068	CACATAGTTTAAAAAACGTTAAGCCAGAAGATACTCCAGGACCTGTATTATTACCAGATAATCATTATTTAAG
CVO069	GGACctcgagTCAGTGATGGTGATGGTGATGTTTATATAATTCATCCATTCCTAAAGTAATACC
CVO071	GGACgaattcATAGCCATCACAAATAAAACTGTAGGAAC
CVO079	AACGAACATTAAGCTGCCATATCC
CVO083	GGATAACTTCGTATAATGTATGCTATACGAAGTTATCGTTCTTAAATTTACTGCTGCATTTTCTAAACTCC
CVO093	GGACgaattcATGATTTTAAAAAAAAGCAAACTGCTTGCACTC
CVO097	CATTTAAACCTTTCCAATATCACACATTTGC
CVO104	GGTTTCCCCCTCCTTTTCAGC
CVO121	GCACccgcggTGGGTAGAGGCAGAAAAAATTCTCG
CVO122	GCACccgcggTCAGAAGATGCTGGTAACGATAACGTAAATTTTTTTG
CVO123	GCACcccgggCGAGATGGACGACGCAAATGGATGAGTCTGC
CVO150	ATTTTATATGCTTTAGGTTATTCGTAGACAGTG
CVO162	CTTGGTACGCAGGTTCACAGCAG
CVO163	ttacaaaatgcttaaGGTGAGACAAGTATAGATTTAAACTATTTTGGCTGG
CVO183	ttacaaaatgcttaaCACATGCGCATTTTCACCAATTTTTGCC
CVO237	GCACccgcggTCAAATTTTTTTGAAAAAATTGACATTGGTGGTCCAAATG
CVO238	GCACccgcggTCAATTGGTGGTCCAAATGAAGTAGAAGG
CVO239	GCACccgcggTCAGAAGTAGAAGGGGTCCACGAAG
CVO240	CGAAGATGACGCGGAGGACATCCATTCCCGCCAATCCACCAGC
CVO241	GCTGGTGGATTGGCGGGAATGGATGTCCTCCGCGTCATCTTCG
CVO254	ATAACTTCGTATAATGTATGCTATACGAAGTTATTTAGTCCTTGTTGAAGAAGATGCCGAAGATCTTC
CVO305	GGACTAAATAACTTCGTATAGCATACATTATACGAAGTTATGCAAATGTGTGATATTGGAAAGG
CVO306	CGAAGTTATGCAAATGTGTGATATTGGAAAGGTTTAAATGAAAATATGTAAATCG
CVO321	GCCTGAGGACACCCCCAACACC
CVO426	GGACaagcctTTAGTGGTGGTGGTGGTGGTG AAATATTCCTTCAAAAAAGTTAACGTTGATGATCC
CVO427	GGACaagcttTTAGTGGTGGTGGTGGTGGTG AAATATTTTTTTAAAAAAGTTAACGTTGATTCCTTC
CVO428	GGACaagcttTTAGTGGTGGTGGTGGTGGTGGATTCCTTCTAAATTATAAGGGCCATCAACAAATATC
CVO451	GGACaagcttTTAGTGGTGGTGGTGGTGGTGAAATATTCCTTCAAAAAAGTTAACGTTGATCTGGCT
RJH19	GAAGGAATATTTGGTATATTTTTTAATAAGGACTAA
RJH20	AAAAAATATACCAAATATTCCTTCAAAAAAGTTAACGTTGATGATCCATAAATTATAAGGGCC
RJH21	AGCCAGATCAACGTTAACTTTTTTAAAAAAATATTTGGTATATTTTTTAATAAGGAC
RJH22	TATTTTTTTAAAAAAGTTAACGTTGATCTGGCTTAAATTATAAGGGCCATCAACAAATATCATTTTAATAAA
RJH23	GGACaagcttCTAGTGATGGTGATGGTGATGGATGATCCATAAATTATAAGGGCCATCAACAAA
RJH24	GGACaagcttCTAGTGATGGTGATGGTGATGTAAATTATAAGGGCCATCAACAAATATCATTTTAATAAAACC
RJH40	GTCCCCGCGGTTAGAAAATGCTGGTAACAATCACATATATCTTCTTAAA
RJH41	GAGAATTTTTTCTGCCTCTACCCAAAAAATAAAAACAGTTATGAGAAGGGCCTGGAT
RJH42	CTTCTCATAACTGTTTTTATTTTTTGGGTAGAGGCAGAAAAAATTCTCGTAATCACGTAT
PFA0210 5′-23	GGACgaattcGATAAAGAGAATACAGCAAGTTTTATAAAAACTGGT
PFA0210 3′-26	GGACctcgagTTAGTGGTGGTGGTGGTGGTGGTCCTTATTAAAAAATATACCAAATATTTTTTTAAAAAAGTTAAC

Generation of the inducible PFA0210c-LoxP strain in the 1G5 background was conducted using the CRISPR/Cas9 system. Schizonts of the 1G5DC strain were transfected according to standard protocol (38) with pBLD529 (the plasmid that introduces the LoxP sites, see below) and the Cas9-expressing pDC2-cam-Cas9-U6-hDHFR (42) plasmid to which the gene encoding the yeast cytosine deaminase-uracil phosphoribosyltransferase had been added, as well as a sequence encoding guide RNAs specific for PFA0210c. Transfectants were initially selected with 2.5 nm WR99210 and were subsequently treated with 1 μm ancotil to select against parasites carrying the pDC-based plasmid. Ancotil-resistant parasites were cloned by limiting dilution.

Integration of the plasmid and recombination of *PFA0210-LoxP* were determined as for *P. knowlesi*, using primer pairs CVO150 with CVO083 and CVO071 with CVO183 to test the presence of the wild type gene, CVO150 with CVO162 and CVO321 with CVO183 to determine integration of the plasmid. Removal of the gene after rapamycin treatment was determined by PCR using primers CVO001 and CVO097.

To induce Cre recombinase activity, parasites in the early ring stage were incubated at 37 °C in the presence of 10 nm rapamycin (added from stocks in DMSO) or the equivalent volume of DMSO as control. After 30–60 min, the parasites were washed once and resuspended in growth medium.

Parasitemia was determined by cell counting using a FACSAria fusion flow cytometer. First, ∼2 μl of infected cell culture was fixed with 0.2% glutaraldehyde in PBS for 1 h and subsequently washed with PBS and stored at 4 °C. Prior to counting, the cells were stained with 2 μm Hoechst 33342 in PBS for 30 min. The number of infected erythrocytes per 50,000 erythrocytes was determined.

##### Immunoblotting and Immunofluorescence Imaging

To detect PKH_020910 and PFA0210c by immunoblotting, extracts of uninfected erythrocytes and erythrocytes infected with schizont stage parasites were produced by suspending the cell pellet in 3 volumes of 1× SDS loading dye and separating the proteins on a SDS-12.5% polyacrylamide gel. The proteins were transferred to nitrocellulose, and after blocking the blot with 5% milk in PBS containing 0.05% Tween, the proteins were detected by incubating the blot with the affinity-purified anti-PKH_020910 or anti-PFA0210c antibody at a dilution of 1:5000 or 1:2500, respectively. Antibody binding was visualized by incubating the blot with HRP-linked goat anti-rabbit secondary antibody and developing with Immobilon Western chemiluminescent HRP substrate (Millipore). Spectrin was detected using an anti-spectrin (α and β) mouse monoclonal antibody (Sigma) and an HRP-linked goat anti-rabbit secondary antibody.

Immunofluorescence imaging was performed as described (43). A small aliquot of infected erythrocytes was spun down and resuspended in PBS containing 4% paraformaldehyde and 0.01% glutaraldehyde. After 1 h of agitation at room temperature, the parasitized erythrocytes were pelleted, washed with PBS, permeabilized with 0.1% Triton X-100 for 15 min, washed once more with PBS, blocked with 3% BSA in PBS, and then incubated for 1 h at room temperature in PBS containing 3% BSA and anti-PKH_020910 antibody diluted at 1:5000. The erythrocytes were then washed three times with PBS and subsequently incubated at room temperature in PBS containing 1 μg/ml 4′,6-diamidino-2-phenylindole (DAPI) and Alexa 596-conjugated anti-rabbit antibodies diluted at 1:5000 for 1 h. The erythrocytes were washed three times with PBS, resuspended in a small volume of PBS, and placed on a polyethyleneimine-coated microscope slide. This was covered with a coverslip and sealed with nail polish. Differential interference contrast and fluorescence images were obtained on a Nikon Eclipse Ni, fitted with a Hamamatsu C11440 digital camera. Images were processed in Photoshop. Note that the anti-PKH020730 signal was false colored green.

##### Production of Plasmids

Plasmids used for disrupting the PKH_020910 locus were produced by amplifying the region of PKH_020910 to be targeted (omitting the first 22 codons of the genomic sequence) by PCR. The resulting fragment was cloned into the XmaI and SacII sites of pHH4-MyoA-GFP (32), producing the plasmids pBLD468 (product of primers CVO123 and CVO122, ending at codon 476 (full length)), pBLD481 (product of primers CVO123 and CVO237, ending at codon 468), pBLD482 (product of primers CVO123 and CVO238, ending at codon 461), pBLD483 (product of primers CVO123 and CVO239, ending at codon 456), and pBLD467 (product of primers CVO123 and CVO121, ending at codon 336). Plasmid pRH34, which contains a fusion of the sequence in pBLD467 and a re-codonized version of the remaining coding sequence, was produced by overlapping PCR. The wild type gene (ending at codon 367) was amplified from genomic *P. knowlesi* DNA (using primers CVO123 and RJH42), and the remaining sequence (codons 368–476) was amplified by PCR using a version of the gene codon-optimized for *Escherichia coli* (GeneArt) (using primers RJH41 and RJH40). The two fragments were fused by PCR, using built-in overlapping ends and primers CVO123 and RJH040. Plasmid pBLD484, which lacks codons 433–444, was also made by overlapping PCR, using primers that amplified codons 23–432 (CVO123 and CVO241) and codons 445–476 with overlapping ends (CVO240 and CVO122). The fragments were fused by PCR using the internal overlap and primers CVO123 and CVO122.

Plasmid pBLD529 that was used to replace the native *PFA0210c* gene with a version containing the SERA2 intron containing a *loxP* site was created as follows. A synthetic gene product containing a fusion of wild type sequence and re-codonized sequence was cloned into the pBAD vector. This sequence was fused to 3′ UTR sequence of *PFA0210c* by overlapping PCR using primer pairs CVO001 and CVO254 (combined with CVO305 to add the LoxP site) to amplify the coding region and CVO306 and CVO163 to amplify the 3′ region. The overlapping PCR introduced a *loxP* site immediately following the stop codon. This sequence was cloned into pGEM-T by T-tailing, creating pBLD509. To introduce the *loxP* site contained in the *SERA2* intron, a synthetic DNA fragment was obtained that contained wild type *PFA0210c* sequence fused with *PFA0210c* re-codonized sequence containing a *SERA2* intron in which a *loxP* site was inserted between bp 463 and 464. A SpeI-Tth111I fragment of this fusion was cloned into pBLD509 to give rise to pBLD529.

Plasmids used for protein production in *E. coli* were produced as follows. As the N-terminal region of PFA0210c does not affect the phospholipid transfer activity of PFA0210c (24), it was not included in the recombinant proteins (“full-length” denotes the presence of the entire C-terminal extension). Gene fragments starting at codon 144 in PFA0210c, codon 111 in PKH_020910, and codon 49 in PCHAS_020730 were amplified from genomic DNA using primer pairs PFA0210c 5′-23 and PFA0210c 3′-26 (*PFA0210c*), CVO014 and CVO015 (*PKH_020910*), and CVO059 and CVO060 (*PCHAS_020730*), respectively, and cloned into either the EcoRI (*PFA0210c* and *PKH_020910*) or the XbaI (*PCHAS_020730*) and the SalI site of pMAL c2x (New England Biolabs). Truncated versions of *PFA0210c*, *PKH_020910,* and *PCHAS_020730* were produced using primer pairs CVO022 and CVO021, CVO014 and CVO064, and CVO059 and CVO61, respectively. To produce the serial truncations of PFA0210c, DNA was amplified with primer CVO022 paired with primer CV057 (terminal codon 459, plasmid pBLD413), primer CVO056 (terminal codon 453, plasmid pBLD412) or primer RJH023 (terminal codon 450, plasmid pRH17), and primer RJH024 (terminal codon 447, plasmid pRH18), and the resulting DNA was cloned into the EcoRI and HindIII sites of pMAL c2x. Each 3′ primer contained the sequence of a hexahistidine tag, hence the resulting protein contained the maltose-binding protein at the N terminus and a hexahistidine tag at the C terminus. The fusions of PFA0210c with the C terminus of PCHAS_020730 or the C terminus of GFP were produced by overlapping PCR. The region of PFA0210c encompassing codon 144–418 was amplified from genomic DNA using primers CVO022 paired with CVO065 for the fusion with PCHAS_020730 and CVO067 for the fusion with GFP, with an overlapping tail complementary to the fragment to be fused. The PCHAS_020730 fragment, spanning codons 368–415, was amplified by PCR using genomic *P. chabaudi* DNA as template using primers CVO065 and CVO060. The GFP fragment, spanning codons 194–241, was amplified by PCR using the gene encoding enhanced GFP as template using primers CVO068 and CVO069 and fused to the PFA0210c fragment by overlapping PCR using primers CVO022 and CVO069.

Point mutations were generated by overlapping PCR. The 5′ region was generated using primer CVO022 paired with primer RJH019 (KK-EG) or RJH021 (WI-SQ), whereas the 3′ region was generated using primers RJH020 (KK-EG) or RJH022 (WI-SQ) paired with M13 forward. DNA fragments were fused by PCR using primers CVO022 and M13 forward using the 5′ and 3′ region as template and cloned into pMAL c2x using EcoRI and HindIII.

To introduce these point mutations into truncated genes, the genes were amplified using primers CVO022 paired with CVO426 (terminal codon 459, KK-EG mutation) using pBLD413 (which contains the gene encoding PFA0210c with codon 459 as terminal codon) as template, CVO427 (terminal codon 459, WI-SQ mutation), or CVO428 (terminal codon 453, WI-SQ mutation). To generate the double mutant, primers CVO022 and CVO451 were used, using pBLD413 as template. The product was cloned into pMAL c2x using EcoRI and HindIII.

##### Protein Purification

All proteins were purified from *E. coli* strain BL21(DE3) as fusions with maltose-binding protein at the N terminus and a hexahistidine tag at the C terminus. Protein production was induced in 1-liter cultures by the addition of isopropyl β-d-1-thiogalactopyranoside to 0.5 mm when the culture was at an *A*_600_ of ∼0.5. The bacteria were harvested after an overnight incubation at 18 °C and resuspended in column buffer (20 mm Tris, pH 7.4, 500 mm NaCl, 20 mm imidazole) containing protease inhibitors (Complete EDTA-free Mixture, Roche Applied Science). The bacteria were lysed with a cell disruptor (Constant Cell Disruption Systems), and the lysate was sonicated with a microtip for three 30-s pulses (50% duty cycle, setting 4; Vibracell, Sonics and Materials). Lysates were clarified by centrifugation in a JA25.5 rotor at 9000 × *g* for 30 min. The clarified lysates were mixed with Ni^2+^-nitrilotriacetic acid resin (Qiagen) and incubated at 4 °C for 1 h while rotating. The mixture was poured into a 1.5 × 12-cm chromatography column (Bio-Rad), and the resin was washed with ∼50 column volumes of column buffer. The protein was eluted with 5 column volumes of column buffer supplemented with 250 mm imidazole. The eluate was concentrated to 0.5–1.5 ml using a Vivaspin 15 concentrator (Sartorium Stedim Biotech) and loaded onto a HiLoad 26/60 Superdex 200 prep-grade column equilibrated in standard assay buffer (10 mm HEPES-Na^+^, pH 7.4, 1 mm EDTA, 50 mm NaCl, pH 7.4). Elution of protein was detected through monitoring the UV absorption of the eluate, followed by SDS-PAGE. The fractions containing monomeric protein were concentrated as described above, aliquoted, and snap-frozen in liquid nitrogen. We were unable to purify unaggregated full-length protein containing the Trp-448 and Ile-449 point mutations in five independent attempts; as aggregated protein is not active, this mutant was not included in the analysis.

##### Phospholipid Transfer Assay

Phospholipid transfer activity was measured as described previously (44). Briefly, donor vesicles and acceptor vesicles were produced by mixing 98 mol % phosphatidylcholine and 2 mol % phosphatidic acid or 88 mol % phosphatidylcholine, 2 mol % phosphatidic acid, 10 mol % *N*-lactosyl-phosphatidylethanolamine (all non-radioactive lipids were obtained from Avanti Polar Lipids, Inc.) and a trace of ^14^C-labeled phosphatidylcholine (l-α-dipalmitoyl-phosphatidylcholine; PerkinElmer Life Sciences), respectively. To this mixture, 200 μl chloroform was added, and the mixture was dried under a stream of N_2_ gas until completely dry. The dried lipids were resuspended in standard assay buffer and solubilized in a sonicating water bath (Ultrawave U300H) until the solution became completely translucent. To measure phospholipid transfer, 69 nmol of acceptor vesicles were mixed with 23 nmol of donor vesicles in the presence of 1 mg/ml essentially fatty acid-free bovine serum albumin (Sigma), followed by the addition of protein to a final concentration of 25 μg/ml. The final volume of the reaction was 100 μl. This mixture was incubated at 37 °C for 30 min. To measure total radioactivity in the sample, a small aliquot was removed, and radioactivity was counted using scintillation counting. To the remaining mixture, agglutinin RCA_120_ (lectin from *Ricinus communis*; Sigma) was added to agglutinate the donor vesicles, and the samples were incubated on ice for 30 min, followed by incubation at room temperature for 10 min. The agglutinated donor vesicles were pelleted by centrifugation for 6 min at 13,000 rpm in a microcentrifuge. The radioactivity in the supernatant was then measured using scintillation counting, and the amount of transfer was calculated.

## Author Contributions

R. J. H., A. R., E. K., R. W. M., and C. v. O. conducted the experiments and analyzed the results. M. J. B. provided technical assistance with the protein purification and advice on experimental design. C. v. O. conceived the idea for the project and wrote the manuscript.

## Footnotes

4 The abbreviation used is:

START: StAR-related lipid-transfer.
